# Toxicity Assessment and Antifungal Potential of Copper(II) and Silver(I) Complexes with 1,10-Phenanthroline-5,6-dione Against Drug-Resistant Clinical Isolates of *Cryptococcus gattii* and *Cryptococcus neoformans*

**DOI:** 10.3390/jof11060436

**Published:** 2025-06-06

**Authors:** Lucas Giovanini, Ana Lucia Casemiro, Larissa S. Corrêa, Matheus Mendes, Thaís P. Mello, Lucieri O. P. Souza, Luis Gabriel Wagner, Christiane Fernandes, Matheus M. Pereira, Lais C. S. V. de Souza, Andrea R. S. Baptista, Josué de Moraes, Malachy McCann, Marta H. Branquinha, André L. S. Santos

**Affiliations:** 1 Laboratório de Estudos Avançados de Microrganismos Emergentes e Resistentes (LEAMER), Departamento de Microbiologia Geral, Instituto de Microbiologia Paulo de Góes (IMPG), Centro de Ciências da Saúde (CCS), Universidade Federal do Rio de Janeiro (UFRJ), Rio de Janeiro 21941-901, RJ, Brazil; giovanini@micro.ufrj.br (L.G.); casemiroanalucia@gmail.com (A.L.C.); lari.corsantos01@gmail.com (L.S.C.); mendessilva2604@gmail.com (M.M.); thaispdmello@gmail.com (T.P.M.); lucieri@micro.ufrj.br (L.O.P.S.); mbranquinha@micro.ufrj.br (M.H.B.); 2 Programa de Pós-Graduação em Microbiologia (PPG-Micro), Instituto de Microbiologia Paulo de Góes (IMPG), Centro de Ciências da Saúde (CCS), Universidade Federal do Rio de Janeiro (UFRJ), Rio de Janeiro 21941-901, RJ, Brazil; 3 Departamento de Química, Universidade Federal Santa Catarina (UFSC), Florianópolis 88035-972, SC, Brazil; luis.gabriel.wagner@hotmail.com (L.G.W.); christiane.horn@ufsc.br (C.F.); 4 Chemical Engineering and Renewable Resources for Sustainability (CERES), Department of Chemical Engineering, University of Coimbra, Pinhal de Marrocos, 3030-790 Coimbra, Portugal; matheus@eq.uc.pt; 5 Departamento de Microbiologia e Parasitologia, Centro de Investigação de Microrganismos, Instituto Biomédico, Universidade Federal Fluminense (UFF), Niterói 24210-130, RJ, Brazil; laiscavalcanti@id.uff.br (L.C.S.V.d.S.); andrearegina@id.uff.br (A.R.S.B.); 6 Programa de Pós-Graduação em Microbiologia e Parasitologia Aplicadas, Instituto Biomédico, Departamento de Microbiologia e Parasitologia, Universidade Federal Fluminense (UFF), Niterói 24210-130, RJ, Brazil; 7 Rede Micologia RJ, Fundação de Amparo à Pesquisa do Estado do Rio de Janeiro (FAPERJ), Rio de Janeiro 21941-901, RJ, Brazil; 8 Centro de Pesquisa de Doenças Negligenciadas, Universidade de Guarulhos, São Paulo 07023-070, SP, Brazil; moraesnpdn@gmail.com; 9 Núcleo de Pesquisa em Doenças Negligenciadas, Instituto Científico e Tecnológico, Universidade Brasil, São Paulo 08230-030, SP, Brazil; 10 Chemistry Department, Maynooth University, W23 VP22 Maynooth, Co. Kildare, Ireland; malachy.mccann@mu.ie; 11 Programa de Pós-Graduação em Bioquímica (PPGBq), Instituto de Química (IQ), Universidade Federal do Rio de Janeiro (UFRJ), Rio de Janeiro 21941-909, RJ, Brazil

**Keywords:** coordination compounds, phendione, *Cryptococcus*, *Galleria mellonella*, antifungal, toxicity

## Abstract

The World Health Organization included *Cryptococcus neoformans* and *Cryptococcus gattii* in its priority fungal pathogen list due to their high mortality rates and frequent treatment failures. These facts have driven research toward the discovery of new compounds for the treatment of cryptococcosis. In this study, we investigated the therapeutic potential of two complexes, [Cu(phendione)_3_](ClO_4_)_2_·4H_2_O (Cu-phendione) and [Ag(phendione)_2_]ClO_4_ (Ag-phendione), against drug-resistant clinical isolates of *C. gattii* and *C. neoformans*. Both complexes demonstrated anti-*Cryptococcus* activity, with Cu-phendione exhibiting minimum inhibitory concentration (MIC) values of 6.25 μM for *C. gattii* and 3.125 μM for *C. neoformans*, while Ag-phendione showed an MIC of 1.56 μM for both *Cryptococcus* species. Notably, both Cu-phendione and Ag-phendione complexes exhibited enhanced antifungal activity against reference strains of *C. neoformans* and *C. gattii*. In silico analysis identified both complexes as highly promising, exhibiting good oral bioavailability, high gastrointestinal absorption, and moderate skin permeability. Moreover, neither complex demonstrated toxicity toward sheep erythrocytes at concentrations up to 62.5 μM, with a selectivity index (SI) exceeding 10 for Cu-phendione and 40 for Ag-phendione. In vivo testing using the *Galleria mellonella* model demonstrated that both complexes were non-toxic, with 100% larval survival at concentrations up to 1000 μM and SI exceeding 160 following a single administration. Interestingly, larvae exposed to Cu-phendione at concentrations of 15.6–31.25 μM exhibited a significant increase in the density of hemocytes, the immune cells responsible for defense in invertebrates. Furthermore, multiple treatments with 62.5 μM of complexes caused either no larval mortality, hemocyte alterations, or changes in silk production or coloration, indicating a lack of toxicity. These findings suggest that Cu-phendione and Ag-phendione may serve as promising antifungal alternatives against *Cryptococcus*, with minimal host toxicity.

## 1. Introduction

Social factors, including the growing population of immunocompromised and/or immunosuppressed individuals, combined with climate changes, have contributed to a global rise in fungal infections, such as cryptococcosis [1,2]. In 2020, an estimated 180,000 cases of cryptococcosis were reported globally, leading to approximately 152,000 cases of meningoencephalitis caused by *Cryptococcus* species and resulting in around 112,000 deaths worldwide [1,3]. The primary group affected by cryptococcosis comprises patients with acquired immunodeficiency syndrome (AIDS), who face a mortality rate of 40–60% even with appropriate treatment [3]. Recent data show a significant rise in cryptococcal meningitis cases, particularly in Europe and North America, where cases nearly doubled between 2014 and 2020 (increasing from 7400 to 12,000 cases), largely due to a growing population of immunosuppressed individuals. Similarly, South America experienced an even sharper increase, with cases more than doubling during the same period (from 5300 to 12,000) [1,3]. Compounding this issue, an increasing number of ostensibly healthy immunocompetent individuals have been diagnosed with cryptococcosis in recent decades, predominantly caused by *C. gattii* [4,5,6,7].

In 2022, the World Health Organization (WHO) published a treatment guideline for cryptococcosis, recommending the use of three classes of antifungal agents: polyenes (e.g., amphotericin B), azoles (e.g., fluconazole), and antimetabolites (e.g., 5-flucytosine) [8]. Notably, the guideline excludes drugs like caspofungin, as the *Cryptococcus* genus exhibits intrinsic resistance to the echinocandin class of antifungals [9]. The scarcity of antifungal agents is further compounded by their limited global availability. For instance, flucytosine is not licensed in most countries on the African continent, which bears the highest burden of cryptococcosis cases worldwide [5,10]. The WHO guideline seeks to standardize the treatment of *Cryptococcus* infections to enhance therapeutic efficacy and minimize patient toxicity. This is particularly crucial as amphotericin B and fluconazole, despite their efficacy, are associated with significant toxicity, which can further deteriorate the health of already vulnerable patients [8,11,12]. However, the misuse of antifungals in both clinical settings and agriculture has accelerated the emergence of drug-tolerant fungal isolates, leading to an increasing number of treatment failures [13,14]. Recognizing this growing threat, the WHO in 2022 identified the most critical fungal pathogens requiring urgent research and intervention due to the high mortality rates, treatment challenges, and the rising prevalence of antifungal resistance. *C. neoformans* was ranked first as a “critical priority” pathogen, while *C. gattii* was categorized as medium priority, ranking 16th on the list. This underscores the urgent need to develop new and effective anti-*Cryptococcus* therapies [15].

Over recent decades, numerous coordination compounds (complexes) have demonstrated substantial antifungal activity [16,17,18,19]. Among these, metal derivatives of the ligand 1,10-phenanthroline-5,6-dione (phendione) complexed with Cu^2+^ or Ag^+^ ions—forming [Cu(phendione)_3_](ClO_4_)_2_·4H_2_O (Cu-phendione) and [Ag(phendione)_2_]ClO_4_ (Ag-phendione), respectively—have emerged as promising antifungal candidates. These complexes exhibit potent antifungal activity against medically significant fungi, such as various *Candida* species [17,20,21,22,23] and filamentous fungi, including *Phialophora verrucosa* [24,25] and the multidrug-resistant species belonging to the *Scedosporium*/*Lomentospora* genera [18,26]. In addition to their antifungal efficacy, Cu-phendione and Ag-phendione have shown low toxicity in various in vitro models, including pulmonary, renal, and hepatic epithelial cells, peritoneal and pulmonary macrophages, and lung fibroblasts [17,23,25,27]. Their safety has also been demonstrated in in vivo models, such as *Galleria mellonella* larvae [17,25,27], Swiss mice [27], and golden hamsters [28]. These findings highlight their potential as effective and safe antifungal agents.

With these considerations in mind, we aimed to determine the minimum inhibitory concentration (MIC) and minimum fungicidal concentration (MFC) of the complexes Cu-phendione and Ag-phendione against six drug-resistant clinical isolates and four reference strains of *C. neoformans* and *C. gattii*. Additionally, we assessed the in silico pharmacokinetic properties and drug-likeness of these complexes using SwissADME software, as well as their stability over a two-year period. Finally, we evaluated the in vitro hemolytic activity and investigated the acute and chronic in vivo toxicity of Cu-phendione and Ag-phendione using the *G. mellonella* larvae model.

## 2. Materials and Methods

### 2.1. Fungi and Growth Conditions

Six clinical isolates belonging to the *Cryptococcus* genus were obtained from patients who attended the Instituto Nacional de Infectologia Evandro Chagas (INI), Fundação Oswaldo Cruz (FIOCRUZ), and were kindly provided by Dr. Rodrigo Almeida-Paes (INI-FIOCRUZ). To comply with ethical standards and institutional agreements between UFRJ and FIOCRUZ, patient data were not disclosed. In this sense, the *C. neoformans* isolates were designated CFP00213 (213), CFP00223 (223), and CFP00350 (350), while the *C. gattii* isolates were labeled CFP00023 (23), CFP00025 (25), and CFP00027 (27). Before each experiment, an aliquot of the culture was transferred from Sabouraud dextrose agar (2% glucose, 1% peptone, and 0.5% yeast extract) to RPMI 1640 medium (Sigma-Aldrich, St. Louis, MO, USA), pH 7.2, buffered with 0.165 M MOPS (Sigma-Aldrich, St. Louis, MO, USA). RPMI medium induces the expression of capsular polysaccharides, the primary virulence factor of *Cryptococcus* cells. The cultures were incubated at 37 °C for 48 h to promote cell proliferation, stimulate metabolic activation, and enhance capsular development [29]. Yeast cell counts were determined using a Neubauer chamber.

### 2.2. Test Compounds

1,10-Phenanthroline-5,6-dione (phendione), [Ag(phendione)_2_]ClO_4_ (Ag-phendione), and [Cu(phendione)_3_](ClO_4_)_2_·4H_2_O (Cu-phendione) were synthesized by Dr. Christiane Fernandes and Luis Gabriel Wagner (Federal University of Santa Catarina, Florianópolis, SC, Brazil) according to methods previously described in the literature [21,30]. The compounds and their respective salts (AgClO_4_ or Cu(ClO_4_)_2_·6H_2_O) were dissolved in dimethyl sulfoxide (DMSO; Sigma-Aldrich, St. Louis, MO, USA) and stored at room temperature in the dark.

### 2.3. Antifungal Susceptibility Profile of Clinically Available Antifungal Agents

The susceptibility profile was evaluated using the microdilution method in RPMI 1640 medium buffered with 0.165 M MOPS, following the M27-A3 protocol for yeasts established by the Clinical and Laboratory Standards Institute (CLSI) [31]. In 96-well cell culture plates (BIOFIL, Boschring, Germany), RPMI was supplemented with one of the following antifungal agents (Sigma-Aldrich, St. Louis, MO, USA): amphotericin B (0.031–16 µg/mL), caspofungin (0.015–8 µg/mL), fluconazole (0.125–64 µg/mL), flucytosine (0.125–64 µg/mL), or voriconazole (0.031–16 µg/mL). The plates were incubated at 37 °C for 24 h to determine the MIC, defined as the lowest concentration that visually inhibited fungal growth. Each fungal isolate was classified as either wild-type or non-wild-type for each antifungal based on the epidemiological cutoff values provided in CLSI M59 ED3, 2020 [32].

### 2.4. Assessment of the Potential Antifungal Activity of Test Compounds

In this series of experiments, the microdilution method described above was first employed to determine the MIC of the test compounds, including the ligand phendione, the simple salts silver perchlorate (AgClO_4_) and copper perchlorate (Cu(ClO_4_)_2_·6H_2_O), and the complexes Cu-phendione and Ag-phendione, at concentrations ranging from 0.097 to 100 µM. The plates were incubated at 37 °C for 24 h. Following MIC determination, the MFC was assessed by inoculating 10 µL from wells showing no visible fungal growth onto Sabouraud dextrose agar. The MFC value was defined as the lowest concentration that completely inhibited fungal growth after 24 h of incubation at 37 °C [19,33]. To further support and strengthen the evidence of the anti-*Cryptococcus* activity of the Cu-phendione and Ag-phendione complexes, we additionally performed MIC/MFC assays using well-characterized standard reference strains: ATCC 28957, H99, T_1_444 (all *C. neoformans*), and R265 (*C. gattii*) [29].

### 2.5. Chemical Stability of the Test Compounds

To evaluate the stability of the antifungal activity of the test complexes, the MIC assay was repeated every six months over a two-year period using the same Ag-phendione or Cu-phendione solution. The solutions were stored at room temperature and protected from light to maintain their chemical integrity [33].

### 2.6. Prediction of Drug-Likeness and Pharmacokinetics of the Test Compounds

The prediction of drug-likeness and pharmacokinetics for the complexes was performed using the online SwissADME software (http://www.swissadme.ch/, accessed on 1 April 2025) [34]. The chemical structures of Ag-phendione and Cu-phendione were created using Discovery Studio, v20 (Accelrys, San Diego, CA, USA). These chemical structures were then submitted to SwissADME software to evaluate their physicochemical properties and estimate their therapeutic potential and pharmacokinetics. Therapeutic potential was assessed using five different rule-based filters for oral bioavailability prediction: Lipinski [35], Ghose [36], Veber [37], Egan [38], and Muegge [39]. As a comparative control, the therapeutic potential and pharmacokinetics of amphotericin B and fluconazole were also predicted, with their SMILES obtained from the PubChem database (https://pubchem.ncbi.nlm.nih.gov/, accessed on 1 April 2025). Pharmacokinetics were evaluated by predicting the inhibition potential of cytochrome P450 isoenzymes (CYP1A2, CYP2C19, CYP2C9, CYP2D6, and CYP3A4). Additionally, physicochemical properties, including topological polar surface area (TPSA) and the partition coefficient between water and n-octanol (LogP), were calculated to estimate passive gastrointestinal absorption (GIA), blood–brain barrier (BBB) penetration, and P-glycoprotein efflux using the BOILED-Egg model [40]. Molecular size and lipophilicity were also calculated to predict the skin permeability coefficient (Log *K*p in cm/s).

### 2.7. Hemolysis Assay

To evaluate the hemolytic activity of the test compounds, the microdilution method was performed in PBS using 96-well cell culture plates, as described by Evans et al. [41]. PBS containing the test compounds (phendione, Cu-phendione, and Ag-phendione) at varying concentrations (1.9–62.5 µM) was added to the wells. Sheep erythrocytes (Cultilab, Rio de Janeiro, RJ, Brazil) were diluted in PBS to a final concentration of 2% (approximately 2 × 10^8^ erythrocytes) and added to each well. The final volume of each well was 200 μL. A negative control (PBS and blood) and a positive control (PBS, blood, and 1% Triton X-100) were also included. The plates were incubated at 37 °C for 24 h, then centrifuged at 500× *g* for 10 min at 4 °C. Following centrifugation, 100 μL of the supernatant from each well was transferred to a new 96-well microplate, and absorbance was measured using a microplate reader (MULTISKAN SkyHigh; Thermo Scientific, Waltham, MA, USA) at 415 nm [19]. To calculate the final results, the average absorbance of the negative control was subtracted from the absorbance values of the other groups. The average absorbance of the positive control was set as 100% hemolysis, and the percentage hemolysis of the other groups was calculated accordingly. The selectivity index (SI) was determined using the following equation:

SI = erythrocyte CC_50_/*Cryptococcus* MIC.

### 2.8. In Vivo Toxicity of the Test Compounds in Galleria mellonella

In this set of experiments, *G. mellonella* larvae weighing approximately 0.2–0.3 g and displaying clear, uniform coloration were used. The larvae were inoculated (10 µL/larva) using an insulin syringe (BD Ultra-Fine, Franklin Lakes, NJ, USA) at the last left proleg. Survival was monitored by observing the larvae’s response to gentle physical stimuli (touches) on the head and body; larvae that did not respond were considered dead. We also assessed their coloration through visual analysis as an indicator of stress levels. Throughout the experiments, the larvae were maintained at 37 °C [19,42]. Two distinct treatment protocols were designed to evaluate the effects of the test compounds on larval survivability, as detailed below.

#### 2.8.1. Single Treatment (Acute Toxicity)

For the acute toxicity analysis, the test compounds were administered to the larvae in a single inoculation at concentrations ranging from 1000 to 125 μM. Each group consisted of 10 larvae, while the control group received only sterile PBS with 4% DMSO. Following inoculation, the larvae were incubated at 37 °C and monitored every 24 h until complete mortality was observed in all inoculated groups or for a maximum of 7 consecutive days [17,19,27]. The SI was calculated using the following equation:

SI = *G. mellonella* CC_50_/*Cryptococcus* MIC.

#### 2.8.2. Multiple Treatment (Chronic Toxicity)

For the chronic toxicity analysis, larvae were inoculated daily for five consecutive days with the test complexes (Cu-phendione or Ag-phendione) at a high concentration of 62.5 μM. Each group consisted of 10 larvae, while the control group received only sterile PBS with 4% DMSO. Following inoculation, the larvae were incubated at 37 °C and monitored every 24 h until complete mortality was observed in all inoculated groups or for a maximum of 7 consecutive days [17,19,27].

### 2.9. Hemocyte Density Analysis

The hemocyte density in the hemolymph of larvae inoculated with both complexes was assessed to evaluate potential immunological changes in *G. mellonella*. Groups of five larvae were either inoculated with Cu-phendione or Ag-phendione at concentrations ranging from 1000 to 15.6 μM or left untreated. The control group received only PBS with 4% DMSO. To analyze hemocyte density, a ventral caudal incision was made between the last prolegs of each larva using a surgical scalpel, and 10 μL of hemolymph was collected. The hemolymph was then diluted 1:100 in 990 μL of an anticoagulant buffer solution (98 mM NaOH, 186 mM NaCl, 17 mM EDTA, and 41 mM citric acid, pH 4.5). The number of hemocytes in each dilution was subsequently counted using a Neubauer chamber (Thermo Fisher Scientific, Waltham, MA USA) [17,19].

### 2.10. Statistical Analyses

All experiments were performed in triplicate and independently repeated at least three times to ensure reproducibility and reliability of the results. The results were analyzed statistically by Student’s *t*-test or by the analysis of variance one-way ANOVA followed by a Tukey–Kramer post-test (in comparisons between three or more groups). In the case of the survival curve, the log-rank (Mantel–Cox) test was used to indicate statistical significance. In all analyses, *p*-values of 0.05 or less were considered statistically significant. All analyses were performed using the program GraphPad Prism version 8.0 (GraphPad Software, San Diego, CA, USA).

## 3. Results

### 3.1. Susceptibility Profile of Cryptococcus Clinical Isolates to Conventional Antifungal Agents

Firstly, we determined the MIC of six clinical *Cryptococcus* isolates against conventional antifungal agents, including amphotericin B, caspofungin, fluconazole, voriconazole, and flucytosine (Table 1). As expected for the *Cryptococcus* genus, all isolates exhibited intrinsic resistance to caspofungin, with MIC values exceeding 8 µg/mL. For amphotericin B, *C. neoformans* isolate 233 displayed a notably higher MIC (>16 µg/mL), while all other *Cryptococcus* isolates had an MIC of 0.03 µg/mL. The MIC values for azoles varied considerably, ranging from 4 µg/mL to over 64 µg/mL for fluconazole and 0.5 to 16 µg/mL for voriconazole. Likewise, flucytosine susceptibility showed isolate-dependent variability (Table 1). Since the CLSI has not yet established clinical resistance breakpoints for antifungals in the *Cryptococcus* genus, we applied the epidemiological cutoff values [32]. These breakpoints define the highest MIC at which a microorganism is considered wild-type, meaning it has no acquired resistance mechanisms. Based on this criterion, all six *Cryptococcus* isolates exhibited non-wild-type resistance mechanisms for voriconazole. Additionally, *C. neoformans* isolate 223 displayed non-wild-type resistance mechanisms for all antifungals tested. Moreover, *C. neoformans* isolate 350 showed non-wild-type characteristics for flucytosine, while *C. gattii* isolate 23 exhibited the same pattern for voriconazole (Table 1).

### 3.2. Impact of Test Compounds on Fungal Viability

The MIC of the ligand phendione, its complexes Cu-phendione and Ag-phendione, and their respective salts (Cu(ClO_4_)_2_·6H_2_O and AgClO_4_) was determined using the microdilution method. Phendione alone exhibited an MIC of 12.5 μM against all six *Cryptococcus* isolates. Both Cu-phendione and Ag-phendione demonstrated greater antifungal activity than the phendione ligand alone. Cu-phendione exhibited an MIC of 6.25 μM for all *C. gattii* isolates and 3.125 μM for all *C. neoformans* isolates. Ag-phendione displayed the highest antifungal potency, with an MIC of 1.56 μM against all *Cryptococcus* isolates. Additionally, the copper salt Cu(ClO_4_)_2_·6H_2_O showed no antifungal activity at any tested concentration, whereas the silver salt (AgClO_4_) exhibited an MIC of 1.56 μM (Table 2). Subsequently, the MFC of the test compounds was determined. Phendione exhibited an MFC ranging from 100 μM to >100 μM (Table 2). Cu-phendione showed an MFC of 12.5 μM against *C. gattii* isolates and 50 μM against *C. neoformans* isolates. Ag-phendione demonstrated potent fungicidal activity, with an MFC of 12.5 μM for all *C. gattii* isolates and 25 μM for all *C. neoformans* isolates. In contrast, the silver salt displayed an MFC of 12.5 μM against all *C. gattii* isolates but exceeded 100 μM for all *C. neoformans* isolates (Table 2).

MIC assays were also conducted using well-characterized reference strains of *C. neoformans* (ATCC 28957, H99, and T1444) and *C. gattii* (R265), which are widely employed in antifungal susceptibility studies. Notably, both Cu-phendione and Ag-phendione complexes exhibited greater antifungal activity against all reference strains (Table 3) compared to the clinical isolates tested (Table 2). These findings not only reinforce the antifungal potential of the complexes, but also add robustness and broader relevance to their activity spectrum against *Cryptococcus* species. This enhanced activity against standardized strains suggests consistent efficacy and supports further exploration of these compounds as promising antifungal agents.

### 3.3. Assessment of the Stability of Antifungal Activity in the Evaluated Complexes

In order to evaluate the long-term stability of the biological activity of the complexes, the MIC assay was repeated every six months over a two-year period using the same solution, which was stored at room temperature and protected from light. The results demonstrated that both Cu-phendione and Ag-phendione exhibited excellent chemical stability, as their antifungal activity remained consistent over time. No variation in MIC values was observed against either *C. neoformans* or *C. gattii*, indicating that the complexes retained their ability to inhibit fungal growth throughout the study period (730 days).

### 3.4. Prediction of Drug-Likeness and Pharmacokinetics of Test Complexes

The SwissADME software evaluates key pharmacokinetic parameters—absorption, distribution, metabolism, and excretion (ADME)—to identify factors that may impede a compound’s ability to reach its biological target [34]. In this context, drug-likeness was assessed using five rule-based filters, where minimal or no violations are preferred. Ag-phendione exhibited a single violation of Lipinski’s rule of five due to its molecular weight exceeding 500 Da [35]. Likewise, it breached the Ghose filter because its molecular weight surpasses the 480 Da threshold [36]. In contrast, Ag-phendione fully complies with both the Veber and Egan filters, showing no violations [37,38]. However, the compound triggered one violation under the Muegge filter, attributed to the presence of more than seven aromatic rings [39] (Table 4). Cu-phendione exhibited two violations of Lipinski’s rule of five, attributed to its molecular weight exceeding 500 Da and the presence of more than 10 nitrogen and oxygen (N or O) atoms [35]. Similarly, the Ghose filter flagged two violations: a molecular weight above 480 Da and a molar refractivity exceeding 130 [36]. While Cu-phendione met the criteria of the Veber filter [37], it violated the Egan filter due to a topological polar surface area (TPSA) greater than 131.6 [38]. Additionally, the Muegge filter identified two violations: molecular weight surpassing 600 Da and the presence of more than seven aromatic rings [39] (Table 4).

For comparison, standard antifungals were also evaluated. Fluconazole exhibited no violations across any of the drug-likeness filters. In contrast, amphotericin B showed extensive non-compliance, with a total of 12 violations across multiple filters. Specifically: (i) Lipinski’s rule flagged three violations—molecular weight exceeding 500 Da, more than ten nitrogen and oxygen (N or O) atoms, and over five hydroxyl (OH) or amine (NH_3_) groups [35]; (ii) the Ghose filter identified three violations—molecular weight greater than 480 Da, molar refractivity exceeding 130, and more than 70 atoms (excluding hydrogens) [36]; (iii) both the Veber and Egan filters flagged one violation each, due to the polar surface area exceeding 140 Å^2^ [37,38]; and (iv) the Muegge filter detected four violations—molecular weight over 600 Da, polar surface area above 150 Å^2^, more than ten hydrogen bond acceptors, and more than five hydrogen bond donors [39] (Table 4).

Based on these findings, both complexes exhibited specific filter violations, indicating potential challenges to their oral bioavailability. Cu-phendione showed a slightly higher number of violations compared to Ag-phendione, suggesting that its molecular properties may impose greater limitations as a drug candidate. Nevertheless, both complexes demonstrated fewer violations than amphotericin B, reflecting a comparatively more favorable drug-likeness profile.

The pharmacokinetic profiles and drug-likeness of Ag-phendione, Cu-phendione, amphotericin B, and fluconazole were also evaluated based on key parameters, including gastrointestinal absorption (GIA), blood–brain barrier (BBB) permeability, P-glycoprotein (P-gp) substrate potential, and inhibition of major cytochrome P450 (CYP) isoenzymes. Skin permeability was also assessed using the logarithm of the skin permeability coefficient (Log *K*p). A detailed summary of these pharmacokinetic properties and drug suitability for each compound is presented in Table 5.

Ag-phendione demonstrates high GIA, indicating good potential for oral uptake. However, it is not predicted to cross the BBB, suggesting limited central nervous system (CNS) penetration. As a P-gp substrate, Ag-phendione may be subject to efflux from various tissues, which could reduce systemic bioavailability but may also mitigate the risk of neurotoxicity. In terms of cytochrome P450 interactions, Ag-phendione inhibits CYP1A2, CYP2C19, and CYP2C9, while showing no inhibitory effect on CYP2D6 or CYP3A4. Given the critical roles of CYP1A2, CYP2C19, and CYP2C9 in drug metabolism [43], these interactions warrant careful consideration during drug development. Additionally, Ag-phendione exhibits a Log *K*p of −7.97 cm/s, indicating moderate skin permeability and potential suitability for transdermal delivery (Table 5).

Cu-phendione shares several pharmacokinetic properties with Ag-phendione, including high GIA and limited ability to cross the BBB, suggesting minimal CNS penetration. Like Ag-phendione, it is identified as a P-gp substrate, indicating potential efflux from tissues that could affect systemic bioavailability. However, Cu-phendione differs in its interaction with cytochrome P450 enzymes; it does not inhibit CYP1A2, CYP2C19, CYP2C9, CYP2D6, or CYP3A4, suggesting a lower risk of metabolic drug–drug interactions. Additionally, Cu-phendione presents a Log *K*p of −8.21 cm/s, reflecting skin permeability comparable to Ag-phendione and supporting its potential for transdermal administration (Table 5).

Amphotericin B showed no inhibitory activity against the major cytochrome P450 isoenzymes—CYP1A2, CYP2C19, CYP2C9, CYP2D6, or CYP3A4—suggesting a high metabolic clearance potential. It was also identified as a P-gp substrate. Despite this, amphotericin B exhibited low GIA, no ability to cross the BBB, and a Log *K*p of −11.94 cm/s, indicating lower skin permeability compared to both Ag-phendione and Cu-phendione. In contrast, fluconazole displayed selective inhibition of CYP2C19, while showing no inhibitory effect on CYP1A2, CYP2C9, CYP2D6, or CYP3A4. Fluconazole was also classified as a P-gp substrate. Notably, it demonstrated high GIA and a Log *K*p of −7.92 cm/s, indicating better skin permeability than the test complexes. Similar to the other compounds, fluconazole was not predicted to permeate the BBB based on SwissADME analysis (Table 5).

### 3.5. Hemolysis Assay

**Figure 1:**
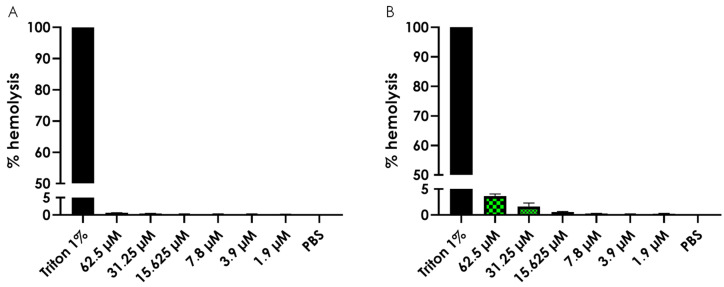
Evaluation of the hemolytic activity of Cu-phendione (**A**) and Ag-phendione (**B**) complexes. Erythrocytes (2%) were incubated for 24 h at 37 °C in the absence or presence of varying concentrations (1.9–62.5 µM) of the test complexes. Hemolysis was quantified as a percentage, with 1% Triton X-100 serving as the positive control (100% hemolysis) and PBS as the negative control (0% hemolysis). Absorbance was measured at 415 nm using an ELISA reader. The hemolysis percentage was calculated by first subtracting the mean absorbance of the negative control from all other absorbance values. The mean absorbance of the positive control was then set as 100% hemolysis, and the relative hemolysis of the test samples was determined accordingly. Data are presented as the mean ± standard deviation of three independent experiments, each performed in triplicate.

To evaluate the potential toxicity of the test complexes, we assessed the hemolytic activity of Cu-phendione and Ag-phendione using a spectrophotometric assay. The results indicated that neither complex exhibited significant hemolytic activity (Figure 1), even at concentrations up to 10 times higher than their respective MIC values.

Based on these results, we calculated the SI values. Since the compounds did not induce hemolysis at the tested concentrations, we assumed the CC_50_ to be higher than the highest concentration tested (>62.5 µM). Consequently, Cu-phendione demonstrated an SI > 10 for *C. gattii* and SI > 20 for *C. neoformans*, while Ag-phendione exhibited an SI > 40 for both fungal species (Table 6).

### 3.6. In Vivo Toxicity of Test Complexes in G. mellonella

#### 3.6.1. Acute Toxicity (Single Dose Treatment)

**Figure 2:**
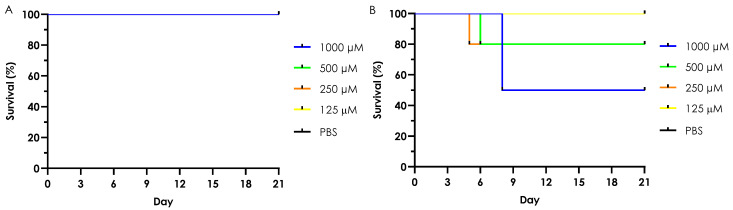
The graphics illustrate the acute toxicity profile of *G. mellonella* larvae inoculated with phendione ligand, Cu-phendione, Ag-phendione, Cu(ClO_4_)_2_.6H_2_O (all sharing the same survival curve as shown in graphic (**A**)), and AgClO_4_ (**B**) at concentrations ranging from 1000 µM to 125 µM. As a control, larvae were inoculated with a PBS + 4% DMSO solution (PBS). The larvae (ten per group) were inoculated with 10 µM of the respective solution using an insulin syringe in the last left proleg and maintained at 37 °C under daily monitoring for a 21-day period.

In this experimental setup, the acute in vivo toxicity of the Cu-phendione and Ag-phendione complexes was evaluated using the *G. mellonella* larvae model, alongside the phendione ligand and their respective salts, AgClO_4_ and Cu(ClO_4_)_2_·6H_2_O. As shown in Figure 2A, none of the tested compounds—phendione, Cu-phendione, Ag-phendione, or simple copper salt—exhibited toxicity at any concentration, with 100% larval survival observed across all groups. However, the silver salt displayed dose-dependent toxicity, causing 50% mortality at the highest tested concentration (1000 µM) and 20% mortality at 500 µM and 250 µM (Figure 2B). Importantly, larvae inoculated with the complexes displayed no visible changes in melanization or silk/cocoon production, which are two physiological markers commonly used to assess stress responses in this model. These observations suggest that the tested complexes do not trigger sub-lethal stress reactions, further supporting their low-toxicity profile [44].

The SI for the toxicity of the complexes in *G. mellonella* larvae was also calculated, assuming a CC₅₀ value greater than 1000 µM for both complexes. Under these conditions, Cu-phendione exhibited an SI greater than 160 against *C. gattii* and greater than 320 against *C. neoformans*. In comparison, Ag-phendione displayed an SI exceeding 641 for both fungal species (Table 6).

#### 3.6.2. Chronic Toxicity (Multiple Dose Treatment)

**Figure 3:**
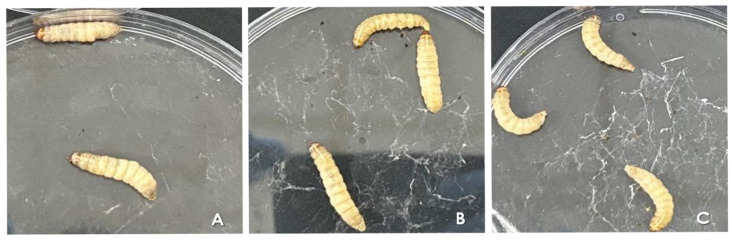
*Galleria mellonella* larvae after five days of daily inoculation with PBS + 4% DMSO (**A**), 62.5 µM Cu-phendione (**B**), or 62.5 µM Ag-phendione (**C**), maintained at 37 °C throughout the experiment. All larvae exhibited a light coloration, as well as normal weight and size, indicating a healthy physiological status.

Chronic toxicity was further evaluated by daily inoculating *G. mellonella* larvae with Cu-phendione and Ag-phendione at 62.5 µM for five consecutive days. Larval survival was monitored over a seven-day period, confirming that both complexes were non-toxic, even under prolonged exposure. Additionally, no alterations were observed in key stress indicators, such as melanization or cocoon formation (Figure 3). These results underscore the resilience of *G. mellonella* larvae and reinforce their suitability as a model for assessing the chronic effects of low-toxicity compounds.

### 3.7. Hemocyte Density Analysis

**Figure 4:**
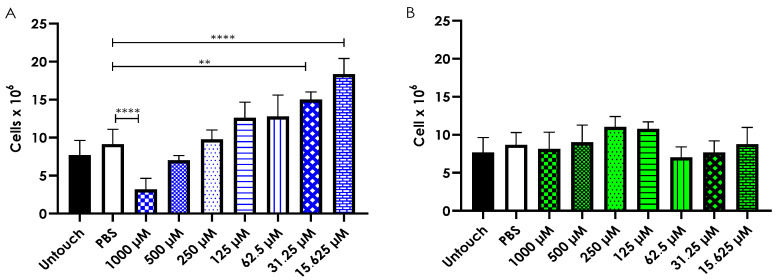
Hemocyte density assay of *Galleria mellonella* larvae, inoculated or not with 10 µL of the complexes Cu-phendione (**A**) or Ag-phendione (**B**) (at concentrations ranging from 1000 µM to 15,625 µM). As a control, we used non-inoculated larvae (untouch) or larvae inoculated with 10 µL of a PBS + 4% DMSO solution (PBS). The larvae (five per group) were inoculated using an insulin syringe in the last left proleg and maintained at 37 °C. Hemolymph collection (10 µL per larva) was performed 24 h after inoculation using a surgical scalpel, diluted in 990 µL of anticoagulant buffer (as described in the methodology), and hemocytes were counted using a Neubauer chamber. Symbols indicate a *p*-value between between 0.009 and 0.0009 (**) and< 0.0001 (****) (one-way ANOVA, Dunnett’s multiple comparison test), denoting the significance of differences in hemocyte counts obtained using a Neubauer chamber from larvae subjected to different treatments. Results are presented as the mean ± standard deviation of three independent duplicates, with each counted twice.

To further explore the in vivo effects of the test complexes on a multicellular organism, hemocyte density, which is the primary immune cells of *G. mellonella*, was assessed following a single inoculation of either Cu-phendione or Ag-phendione. After 24 h, larvae treated with the highest concentration of Cu-phendione (1000 µM) showed a significant reduction in total hemocyte count (Figure 4A). Interestingly, lower Cu-phendione concentrations, specifically 31.25 µM (*p* = 0.0011) and 15.625 µM (*p* < 0.0001), resulted in a significant increase in hemocyte density compared to PBS-inoculated controls. No notable changes in hemocyte levels were observed at the other tested concentrations of Cu-phendione. In contrast, Ag-phendione treatment did not significantly affect hemocyte density at any concentration tested (Figure 4B).

**Figure 5:**
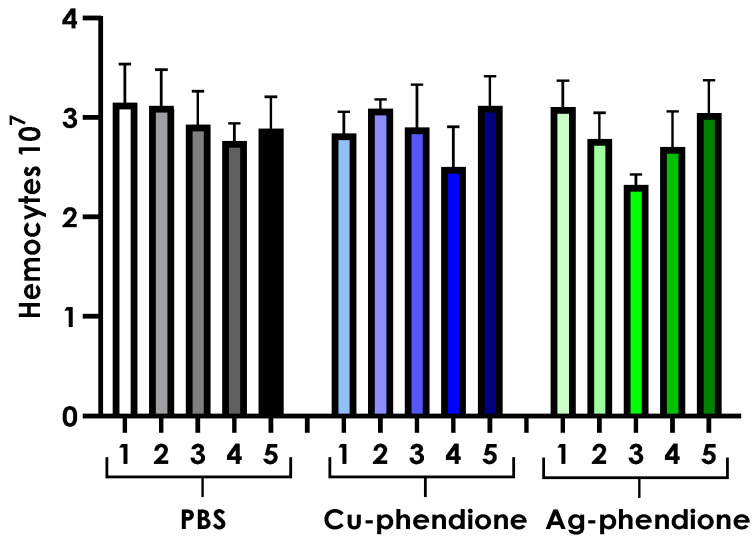
Hemocyte density (at a concentration of 10^7^ cells) from hemolymph samples collected daily over five days (1, 2, 3, 4, and 5) from *Galleria mellonella* larvae inoculated daily (from day 0 to day 4) with 10 µL containing 62.5 µM of Cu-phendione or Ag-phendione, or with a PBS + 4% DMSO solution (PBS). The larvae were maintained at 37 °C and monitored daily throughout the entire experiment (until day 5). The hemolymph was collected (10 µL per larva) using a surgical scalpel, diluted in 990 µL of anticoagulant buffer (as described in the methodology), and hemocytes were counted using a Neubauer chamber.

Hemocyte density was also evaluated in larvae subjected to daily inoculations of 62.5 µM Cu-phendione or Ag-phendione for five consecutive days. Chronic exposure to either complex did not result in significant changes in hemocyte density compared to PBS-inoculated controls (Figure 5).

## 4. Discussion

The limited efficacy and high toxicity of current antifungal agents highlight the urgent need for novel therapeutics, particularly against cryptococcosis [1,15,45]. Our previous studies have demonstrated the potent antifungal properties of Cu-phendione and Ag-phendione complexes against both filamentous fungi, including *Phialophora verrucosa*, *Scedosporium apiospermum*, *Scedosporium minutisporum*, *Scedosporium aurantiacum*, and *Lomentospora prolificans* [18,24,25,26], as well as yeast-like fungi such as *Candida albicans, Clavispora lusitaniae* (formerly *Candida lusitaniae*), *Candida tropicalis, Meyerozyma guilliermondii* (formerly *Candida guilliermondii*), *Kluyveromyces marxianus* (formerly *Candida kefyr*), *Pichia kudriavzevii* (formerly *Candida krusei*), *Candida dubliniensis*, *Debarymyces hansenii* (formerly *Candida famata*), *Nakaseomyces glabratus* (formerly *Candida glabrata*), *Candida parapsilosis, Candida haemulonii*, *Candida haemulonii* var. *vulnera*, and *Candida duobushaemulonii* [17,20,21,22,23]. These findings position metal-based compounds as promising candidates for antifungal drug development. In this study, Cu-phendione and Ag-phendione also exhibited potent activity against drug-resistant *Cryptococcus* clinical isolates, including *C. neoformans* and *C. gattii*, which display non-wild-type phenotypes and resistance to amphotericin B, fluconazole, voriconazole, and flucytosine.

A comparative analysis of the antifungal activity of the complexes and the ligand phendione revealed that Ag-phendione exhibited the strongest anti-*Cryptococcus* activity (in both MIC and MFC parameters), followed by Cu-phendione, with phendione being the least effective. This activity trend (Ag-phendione > Cu-phendione > phendione) was consistent with previous findings against other fungi, such as *Candida* spp. [20,22] and *P. verrucosa* [24]. Notably, while the copper salt showed no anti-*Cryptococcus* activity, the silver salt exhibited an MIC comparable to that of Ag-phendione. A similar trend was observed against other clinically relevant yeast species, including *Candida* and phylogenetically related genera [20,22,23]. However, despite its well-documented antifungal properties, silver salt is known for its toxicity in humans and other mammals [46,47,48], limiting its use primarily to topical applications [46,48,49]. Given its potent antimicrobial activity, researchers have explored the synthesis of silver-based coordination compounds to enhance therapeutic potential while mitigating toxicity concerns [50,51,52].

The literature reports that complexes of gold [16], copper [53,54], vanadium [54], cobalt [55], iridium [56], ruthenium [57,58], rhodium [57], zinc, palladium [59], manganese, nickel [53], bismuth [60], and silver [61] have been synthesized and tested against *Cryptococcus*. A comparative analysis of these complexes revealed that Cu-phendione exhibited a lower MIC value than 44 of the 47 reported compounds, with the exception of one cobalt-based and two gold-based phosphine-linked compounds [16,55]. Similarly, only two compounds demonstrated activity comparable to Ag-phendione: a cobalt-based complex ([Co(di-acetyl thiosemicarbazone)(4-(dimethylamino)pyridine)_2_]NO_3_) [55] and a gold-based complex (Bis-[1,2-bis[(2S,5S)-2,5-dimethylphospholano]benzene]gold(I)) [16]. Additionally, another gold-based compound (Bis-[1,2-bis[(2R,5R)-2,5-dimethylphospholano]benzene]gold(I)) exhibited even greater activity than Ag-phendione [16]. Although Au⁺-phosphine formulations are more effective than Cu-phendione and Ag-phendione, with MIC values around 0.6–1.2 μM [16], the latter compounds offer a more cost-effective alternative, making them a viable option for potential commercial development.

Given the potent anti-*Cryptococcus* activity of Ag-phendione and Cu-phendione, we assessed their therapeutic and pharmacokinetic potential. Ag-phendione violated three rules based on the applied filters but remained within acceptable limits across all five criteria [34]. In contrast, Cu-phendione violated seven rules under the same evaluation. However, both complexes showed significantly fewer violations compared to amphotericin B, which exhibited 12 violations and exceeded the permissible limits for all five filters. Despite its widespread clinical use against fungal infections [62], amphotericin B presented nine more violations than Ag-phendione and five more than Cu-phendione. Thus, while both metal-based complexes have some rule violations, they remain promising antifungal candidates with better pharmacokinetic profiles than amphotericin B.

Both Ag-phendione and Cu-phendione exhibit moderate skin permeability, high gastrointestinal absorption (GIA), and no permeability through the blood–brain barrier (BBB), while also serving as substrates for P-glycoprotein. These properties suggest their potential for transdermal administration, depending on skin conditions and formulation, or oral administration due to the efficient absorption in the gastrointestinal tract. Additionally, their systemic distribution and excretion through various tissues may help reduce treatment toxicity [63,64]. However, while P-glycoprotein efflux may lower toxicity, it could also limit bioavailability in certain tissues [63,64]. If further studies confirm that Cu-phendione and Ag-phendione do not cross the BBB, their therapeutic potential for cryptococcal meningitis may be limited [65,66]. Notably, although amphotericin B and fluconazole are the primary antifungals for cryptococcal meningitis [8], Swiss ADME software also predicts they lack BBB permeability. This discrepancy arises because BBB integrity is compromised during cryptococcal meningitis, allowing antifungal agents to penetrate the meninges [67,68]. Thus, while in silico analysis suggests that Cu-phendione and Ag-phendione do not cross the BBB, future in vivo studies may reveal their potential efficacy in treating cryptococcal meningitis.

Cu-phendione and Ag-phendione exhibit distinct inhibitory effects on cytochrome P450 isoenzymes. Theoretical analysis suggests that Cu-phendione does not inhibit CYP1A2, CYP2C19, CYP2C9, CYP2D6, or CYP3A4, indicating a lower risk of drug–drug interactions involving these enzymes. In contrast, Ag-phendione inhibits CYP1A2, CYP2C19, and CYP2C9 but does not affect CYP2D6 or CYP3A4. Since CYP3A4 metabolizes approximately 50% of marketed drugs and is predominantly expressed in hepatocytes [69,70], the activity of CYP2D6 and CYP3A4 may be sufficient for Ag-phendione metabolism. While isoenzyme inhibition can slow Ag-phendione metabolism and excretion, potentially leading to toxicity, it may also enhance drug accumulation and prolong therapeutic exposure [63,70]. Notably, Cu-phendione and Ag-phendione exhibit absorption levels comparable to fluconazole and higher than amphotericin B, with metabolism and excretion profiles similar to both conventional antifungals. If rapid metabolism and excretion limit their therapeutic potential, nanocarrier strategies such as liposomes, micelles, or micelle-encapsulated liposomes could offer an effective solution. Lipid-based nanocarriers improve drug stability and bioavailability while reducing host toxicity, ultimately enhancing therapeutic efficacy [71].

Once a drug enters the bloodstream, it may interact with various blood components, potentially causing adverse effects such as erythrocyte lysis, the destruction of red blood cells, which can lead to anemia. Therefore, evaluating the hemolytic potential of any candidate drug intended for systemic use is essential [72,73,74]. To assess the toxicity of Cu-phendione and Ag-phendione, we measured their hemolytic activity in vitro using sheep erythrocytes as a model. Our results showed that neither compound induced hemolysis at the tested concentrations (up to 62.5 μM), indicating a favorable safety profile. However, while sheep erythrocytes are commonly used in in vitro assays, they exhibit structural differences [75] and distinct responses to oxidative stress [76] compared to human erythrocytes. Despite these differences, this model serves as a valuable preliminary screening tool for estimating cytotoxicity in mammalian red blood cells. Nonetheless, further toxicity assays using human erythrocytes are necessary to confirm these findings.

To reduce the use of mammals in in vivo assays, *G. mellonella* has emerged as a simple and promising alternative. This model offers several advantages, including easy handling without the need for specialized infrastructure, low maintenance costs compared to mammalian models, and an innate immune system similar to that of mammals. Additionally, *G. mellonella* larvae can thrive at 37 °C, do not require ethical committee approval, and have a rapid reproductive cycle, allowing for high-throughput experimentation and simultaneous evaluation of multiple systems [77,78]. For these reasons, *G. mellonella* has been widely used as a preliminary in vivo model to assess the toxicity and efficacy of novel drug treatments [77,78,79].

The toxicity of other 1,10-phenanthroline- and phendione-based compounds has been evaluated in *G. mellonella*. Gandra et al. [17] reported that several compounds, including {[Cu(3,6,9-trioxaundecanedioic acid)(1,10-phenanthroline)_2_]_3_H_2_O·EtOH}_n_, [Mn(phthalic acid)(1,10-phenanthroline)(H_2_O)_2_], and [Ag(1,10-phenanthroline)_2_]ClO_4_, exhibited no toxicity in *G. mellonella* larvae within 72 h when inoculated at concentrations up to 500 µg/mL. However, at 750 µg/mL, some compounds, such as {[Cu(3,6,9-trioxaundecanedioic acid)(1,10-phenanthroline)_2_]_3_H_2_O·EtOH}_n_, [Mn(phthalic acid)(1,10-phenanthroline)(H_2_O)_2_], and {[Mn(1,10-phenanthroline)_2_(H_2_O)_2_]}_2_(isophthalic acid)_2_(1,10-phenanthroline)·12H_2_O, induced mortality rates of 60%, 40%, and 13%, respectively [17]. The remaining compounds tested showed no toxicity at this concentration. Notably, Cu-phendione and Ag-phendione exhibited at least 160-fold higher fungal selectivity than their MIC values against *Cryptococcus* clinical isolates, underscoring their potential as promising antifungal candidates to address the global shortage of effective treatments.

Some antifungals not only inhibit pathogens but also enhance the antimicrobial response through immunomodulation. For example, amphotericin B promotes a pro-inflammatory response, aiding pathogen elimination [80]. Similarly, certain compounds can increase hemocyte density—the immune cells of *G. mellonella*—either by recruiting cells or stimulating their proliferation and activation, suggesting a pro-inflammatory immunomodulatory effect [17,81,82]. Conversely, a reduction in hemocyte density may indicate a toxic effect [17,81]. To investigate a potential pro-inflammatory effect, we analyzed hemocyte density in larvae 24 h after acute or chronic inoculation with Cu-phendione or Ag-phendione. Acute Ag-phendione inoculation did not alter hemocyte density. However, Cu-phendione exhibited a significant increase, consistent with findings by Gandra et al. [17], who observed a similar effect in larvae inoculated with 750 µg/mL of manganese-based complexes with 1,10-phenanthroline. Additionally, Gandra et al. [17] demonstrated that certain 1,10-phenanthroline- or phendione-based complexes modulate the immune response in *G. mellonella*, including [Mn_2_(isophthalic acid)_2_(1,10-phenanthroline)_3_]·4H_2_O, [Mn(terephthalic acid)(1,10-phenanthroline)_2_]·5H_2_O, {[Mn(3,6,9-trioxoundecanedioic acid)(1,10-phenanthroline)_2_]·3H_2_O·EtOH}_n_, and [Ag(1,10-phenanthroline)_2_]ClO_4_. These compounds increased RNA levels encoding antimicrobial peptides such as transferrin and inducible metalloproteinase inhibitor protein (IMPI), while [Mn(terephthalic acid)(phen)_2_]·5H_2_O upregulated *Gallerimycin* expression and [Ag(1,10-phenanthroline)_2_]ClO_4_ increased *Galiomycin* expression. Notably, some antifungals with strong antifungal activity, like Ag-phendione, do not increase hemocyte density, a pattern also observed with caspofungin [81].

To better simulate clinical treatment, we implemented a daily inoculation strategy in *G. mellonella* larvae, allowing us to evaluate the toxicity of chronic treatment with Cu-phendione and Ag-phendione complexes. Remarkably, the larvae exhibited 100% survival following daily inoculations of Cu-phendione or Ag-phendione at concentrations of 62.5 µM for up to five days, the maximum duration of the experiment. We also monitored hemocyte density daily, with no significant differences observed compared to the control group. These results indicate that Cu-phendione and Ag-phendione have extremely low toxicity in the *G. mellonella* model, whether administered as a single dose or through daily dosing. The daily chronic inoculation strategy more closely replicates therapeutic regimens used in clinical practice, offering a more accurate assessment of the biological and antimicrobial activities of these complexes while maintaining drug bioavailability at a predefined concentration. This approach contrasts with acute inoculation strategies [83,84]. Moreover, this new approach, using an invertebrate model, supports the application of the 3Rs principle (replacement, reduction, and refinement), promoting more efficient use of mammals when necessary [85]. Interestingly, to the best of our knowledge, this is the first reported use of daily treatment in *G. mellonella* for testing the toxicity of new drugs. This novel approach further underscores the potential of *G. mellonella* as a reliable model for evaluating the safety and efficacy of emerging drug candidates. By mimicking chronic treatment regimens more closely than traditional acute dosing methods, this model provides a valuable platform for assessing long-term drug effects and toxicity in a manner that better reflects clinical conditions.

The exceptionally low toxicity of Cu-phendione and Ag-phendione observed in our in vitro (hemolytic activity) and in vivo (acute and chronic toxicity in *G. mellonella*) assays is consistent with previous studies in mouse and hamster models [27,28]. In mice, acute toxicity testing revealed 100% tolerance to inoculations of up to 45 mg/kg of Cu-phendione and 150 mg/kg of Ag-phendione. For the chronic toxicity assessment, mice received daily doses of 45 mg/kg of the compounds for five consecutive days, followed by an additional seven days of observation. Throughout this period, no deaths or behavioral, dietary, or body weight changes were noted. At the study’s conclusion, blood samples were collected for analysis of aspartate aminotransferase (AST) and alanine aminotransferase (ALT) activities—common markers of liver stress. No significant differences were observed between the control and treated groups [27]. In hamsters, administration of 1 mg/kg of Cu-phendione or Ag-phendione, five times per week for nine weeks, similarly resulted in no deaths or changes in behavior, diet, or body weight. Upon completion of the study, blood samples were collected to evaluate renal and hepatic function by measuring urea, creatinine, AST, ALT, and alkaline phosphatase levels. Again, no differences were observed between the control and treated groups [28]. These consistent findings across multiple in vivo models further support the safety profile of Cu-phendione and Ag-phendione and highlight their promising potential for therapeutic use.

Previous studies have demonstrated that the antifungal activity of Cu-phendione and Ag-phendione in various fungal genera is linked to the disruption of plasma membrane integrity and mitochondrial dysfunction [20,21]. These effects lead to fungal DNA cleavage [21] and are accompanied by various morphological alterations and/or changes in the expression of surface molecules on treated cells [20,21,26]. Collectively, these findings suggest that the mechanisms of action of these complexes may differ from those of conventional antifungals currently used in clinical practice, thereby offering a wider range of therapeutic targets. Consequently, future studies investigating the potential of combining these complexes with traditional antifungal therapies could provide valuable insights into novel treatment strategies.

## 5. Conclusions

The present study demonstrated the promising antifungal activity of the Cu-phendione and Ag-phendione complexes against *C. neoformans* and *C. gattii*, including potent efficacy against clinical isolates exhibiting reduced susceptibility to amphotericin B and/or fluconazole. Additionally, in silico analysis using the Swiss ADME software revealed that both complexes exhibit good oral bioavailability and can be readily metabolized and excreted by the organism. Their low toxicity was supported by in vitro and in vivo assays, showing that neither compound induces hemolysis in sheep erythrocytes (in vitro) at concentrations of at least 10× MIC, nor do they cause death in *G. mellonella* larvae or reduce their hemocyte density, whether in acute or continuous treatment. These findings highlight the potential of these complexes as alternative therapeutic agents in the face of emerging global antifungal resistance crisis. However, we emphasize that further investigations are essential. Future studies should include a larger and more diverse panel of clinical isolates with varying antifungal susceptibility profiles to robustly confirm and extend these preliminary results. Additionally, in-depth analyses of the underlying mechanisms of action and in vivo efficacy are warranted to fully establish the clinical relevance of these complexes.

## Figures and Tables

**Table 1:** Susceptibility profile of clinical isolates of *C. neoformans* and *C. gattii* to standard antifungals using the CLSI M27-A3 microdilution method.

*Cryptococcus* Species	Isolate	MIC, [µg/mL]				
		Amphotericin B	Caspofungin	Fluconazole	Flucytosine	Voriconazole
*C. neoformans*	213	0.03 (0.03 µM)	>8 (>7.30 µM)	4 (13 µM)	4 (30 µM)	0.5 (1.4 µM) *
	223	>16 (>17.30 µM) *	>8 (>7.30 µM)	>64 (>208 µM) *	64 (495.76 µM) *	16 (45.80 µM) *
	350	0.03 (0.03 µM)	>8 (>7.30 µM)	8 (26.12 µM)	16 (123.94 µM) *	2 (5.72 µM) *
*C. gattii*	27	0.03 (0.03 µM)	>8 (>7.30 µM)	16 (52.24 µM)	4 (30 µM)	4 (11.45 µM) *
	25	0.03 (0.03 µM)	>8 (>7.30 µM)	16 (52.24 µM)	4 (30 µM)	2 (5.72 µM) *
	23	0.03 (0.03 µM)	>8 (>7.30 µM)	32 (104.48 µM) *	0.125 (0.96 µM)	4 (11.45 µM) *

* Isolates with non-wild-type characteristics—characterized by an MIC exceeding the threshold defined by the epidemiological cutoff values [32].

**Table 2:** Susceptibility profile of clinical isolates of *C. neoformans* and *C. gattii* to Cu-phendione and Ag-phendione complexes, their respective salts, and the phendione ligand, determined using the CLSI M27-A3 [31] microdilution method.

*Cryptococcus* Species	Isolate	MIC/MFC, [µM]				
		Phendione	Cu-Phendione	Ag-Phendione	AgClO_4_	Cu(ClO_4_)_2_.6H_2_O
*C. neoformans*	213	12.5/>100	3.125/50	1.56/25	1.56/>100	>100/ND
	223	12.5/100	3.125/50	1.56/25	1.56/>100	>100/ND
	350	12.5/100	3.125/50	1.56/25	1.56/>100	>100/ND
*C. gattii*	27	12.5/>100	6.25/12.5	1.56/12.5	1.56/12.5	>100/ND
	25	12.5/100	6.25/12.5	1.56/12.5	1.56/12.5	>100/ND
	23	12.5/>100	6.25/12.5	1.56/12.5	1.56/12.5	>100/ND

ND, non-determined.

**Table 3:** Susceptibility profile of reference strains of *C. neoformans* and *C. gattii* to Cu-phendione and Ag-phendione complexes determined using the CLSI M27-A3 [31] microdilution method.

*Cryptococcus* Species	Isolate	MIC, [µM]	
		Cu-Phendione	Ag-Phendione
*C. neoformans*	ATCC 28957	1.56	0.19
	H99	0.78	0.78
	T_1_444	0.78	0.78
*C. gattii*	R265	0.19	0.19

**Table 4:** Drug-likeness assessment of Cu-phendione, Ag-phendione, amphotericin B, and fluconazole based on rule violations across multiple filters (Lipinski, Ghose, Veber, Egan, and Muegge).

Filters	Number of Violations			
	Ag-Phendione	Cu-Phendione	Amphotericin B	Fluconazole
Lipinski	1	2	3	0
Ghose	1	2	3	0
Veber	0	0	1	0
Egan	0	1	1	0
Muegge	1	2	4	0

**Table 5:** Pharmacokinetic evaluation of Cu-phendione, Ag-phendione, amphotericin B and fluconazole: CYP450 inhibition, absorption, distribution, and skin permeability.

Pharmacokinetic Parameters	Test Compounds			
	Ag-Phendione	Cu-Phendione	Amphotericin B	Fluconazole
Inhibition of CYP1A2	Yes	No	No	No
Inhibition of CYP2C19	Yes	No	No	Yes
Inhibition of CYP2C9	Yes	No	No	No
Inhibition of CYP2D6	No	No	No	No
Inhibition of CYP3A4	No	No	No	No
Gastrointestinal absorption	High	High	Low	High
Blood-brain barrier permeability	No	No	No	No
P-glycoprotein substrate	Yes	Yes	Yes	Yes
Log *K*p (cm/s)	−7.97	−8.21	−11.94	−7.92

**Table 6:** Selectivity index of Cu-phendione and Ag-phendione based on CC_50_ and MIC values against *C. gattii* and *C. neoformans*.

*Cryptococcus* Species	Cu-Phendione		Ag-Phendione	
	Erythrocytes	*G. mellonella* Larvae	Erythrocytes	*G. mellonella* Larvae
*C. gattii*	>10	>160	>40	>641
*C. neoformans*	>20	>320	>40	>641

## Data Availability

The original contributions presented in this study are included in the article. Further inquiries can be directed to the corresponding author.

## References

[1] Denning D.W. (2024). Global incidence and mortality of severe fungal disease. Lancet Infect. Dis..

[2] Williams S.L., Toda M., Chiller T., Brunkard J.M., Litvintseva A.P. (2024). Effects of climate change on fungal infections. PLoS Pathog..

[3] Zhao Y., Ye L., Zhao F., Zhang L., Lu Z., Chu T., Wang S., Liu Z., Sun Y., Chen M. (2023). *Cryptococcus neoformans*, a global threat to human health. Infect. Dis. Poverty.

[4] Gbadamosi H., Afriyie-Mensah J.S., Ansah E.N., Dadzie S.K., Puplampu P. (2024). A rare case of disseminated pulmonary cryptococcosis in an immunocompetent patient. BMC Pulm. Med..

[5] Lee Y.M., Liu Y.M., Chen T.C. (2024). Disseminated *Cryptococcus neoformans* infection involving multiple bones and lung in an immunocompetent patient: A case report. BMC Infect. Dis..

[6] Maccora K.A., Tang Y.F., Lee J.H., Chong E.W., Chan H.H.L. (2024). Cryptococcal meningitis with immune-reconstitution inflammatory syndrome causing papilledema and visual field defects in an immunocompetent patient. J. Neuroophthalmol..

[7] Tarisawa M., Kano T., Ishimaru T., Nomura T., Mizushima K., Horiuchi K., Iwata I., Ura S., Minami N., Hozen H. (2024). Clinical characteristics of patients with cryptococcal meningitis in Hokkaido: A case series. Intern. Med..

[8] World Health Organization (WHO) (2022). Guidelines for Diagnosing, Preventing and Managing Cryptococcal Disease Among Adults, Adolescents and Children Living with HIV.

[9] Aguiar T.K., Costa A.C., Neto N.A., Brito D.M., Freitas C.D., Neto J.M., Mesquita F.P., Souza P.F. (2024). Rise and fall of caspofungin: The current status of caspofungin as a treatment for *Cryptococcus neoformans* infection. Future Microbiol..

[10] Riera F., Caeiro J.P., Cornely O.A., Salmanton-García J., Argentinian IFI Diagnostic and Treatment Capacity Group (2023). The Argentinian landscape of mycological diagnostic capacity and treatment accessibility. Med. Mycol..

[11] Abdel-Hafez Y., Siaj H., Janajri M., Abu-Baker Y., Nazzal Z., Hamdan Z., Adwan R., Aiesh B.M., Anaya A.I. (2022). Tolerability and epidemiology of nephrotoxicity associated with conventional amphotericin B therapy: A retrospective study in tertiary care centers in Palestine. BMC Nephrol..

[12] Rakhshan A., Rahmati Kamel B., Saffaei A., Tavakoli-Ardakani M. (2023). Hepatotoxicity induced by azole antifungal agents: A review study. Iran. J. Pharm. Res..

[13] Drakulovski P., Krasteva D., Bellet V., Randazzo S., Roger F., Pottier C., Bertout S. (2023). Exposure of *Cryptococcus neoformans* to seven commonly used agricultural azole fungicides induces resistance to fluconazole as well as cross-resistance to voriconazole, posaconazole, itraconazole and isavuconazole. Pathogens.

[14] Melhem M.S.C., Leite Júnior D.P., Takahashi J.P.F., Macioni M.B., Oliveira L., de Araújo L.S., Fava W.S., Bonfietti L.X., Paniago A.M.M., Venturini J. (2024). Antifungal resistance in cryptococcal infections. Pathogens.

[15] World Health Organization (WHO) (2022). Fungal Priority Pathogens List to Guide Research, Development and Public Health Action.

[16] Dennis E.K., Kim J.H., Parkin S., Awuah S.G., Garneau-Tsodikova S. (2020). Distorted gold(I)-phosphine complexes as antifungal agents. J. Med. Chem..

[17] Gandra R.M., McCarron P., Viganor L., Fernandes M.F., Kavanagh K., McCann M., Branquinha M.H., Santos A.L.S., Howe O., Devereux M. (2020). In vivo activity of copper(II), manganese(II), and silver(I) 1,10-phenanthroline chelates against *Candida haemulonii* using the *Galleria mellonella* model. Front. Microbiol..

[18] Mello T.P., Aor A.C., Barcellos I.C., Pereira M.M., McCann M., Devereux M., Branquinha M.H., Santos A.L.S. (2023). Active Cu(II), Mn(II) and Ag(I) 1,10-phenanthroline/1,10-phenanthroline-5,6-dione/dicarboxylate chelates: Effects on *Scedosporium*. Future Microbiol..

[19] Frota H.F., Lorentino C.M.A., Barbosa P.F., Ramos L.S., Barcellos I.C., Giovanini L., Souza L.O.P., Oliveira S.S.C., Abosede O.O., Ogunlaja A.S. (2024). Antifungal potential of the new copper(II)-theophylline/1,10-phenanthroline complex against drug-resistant *Candida* species. Biometals.

[20] Eshwika A., Coyle B., Devereux M., McCann M., Kavanagh K. (2004). Metal complexes of 1,10-phenanthroline-5,6-dione alter the susceptibility of the yeast *Candida albicans* to amphotericin B and miconazole. Biometals.

[21] McCann M., Coyle B., McKay S., McCormack P., Kavanagh K., Devereux M., McKee V., Kinsella P., O’Connor R., Clynes M. (2004). Synthesis and X-ray crystal structure of [Ag(phendio)_2_]ClO_4_ (phendio = 1,10-phenanthroline-5,6-dione) and its effects on fungal and mammalian cells. Biometals.

[22] Gandra R.M., McCarron P., Fernandes M.F., Ramos L.S., Mello T.P., Aor A.C., Branquinha M.H., McCann M., Devereux M., Santos A.L.S. (2017). Antifungal potential of copper(II), manganese(II) and silver(I) 1,10-phenanthroline chelates against multidrug-resistant fungal species forming the *Candida haemulonii* complex: Impact on the planktonic and biofilm lifestyles. Front. Microbiol..

[23] Gandra R.M., Pacheco C.A., Sangenito L.S., Ramos L.S., Souza L.O., McCarron P., McCann M., Devereux M., Branquinha M.H., Santos A.L.S. (2024). Manganese(II), copper(II) and silver(I) complexes containing 1,10-phenanthroline/1,10-phenanthroline-5,6-dione against *Candida* species. Future Microbiol..

[24] Granato M.Q., Gonçalves D.S., Seabra S.H., McCann M., Devereux M., Santos A.L.S., Kneipp L.F. (2017). 1,10-phenanthroline-5,6-dione-based compounds are effective in disturbing crucial physiological events of *Phialophora verrucosa*. Front. Microbiol..

[25] Granato M.Q., Mello T.P., Nascimento R.S., Pereira M.D., Rosa T.L.S.A., Pessolani M.C.V., McCann M., Devereux M., Branquinha M.H., Santos A.L.S. (2021). Silver(I) and copper(II) complexes of 1,10-phenanthroline-5,6-dione against *Phialophora verrucosa*: A focus on the interaction with human macrophages and *Galleria mellonella* larvae. Front. Microbiol..

[26] Mello T.P., Silva B.A., Lione V., Devereux M., McCann M., Branquinha M.H., Santos A.L.S. (2025). Impact of copper(II) and silver(I) complexes containing 1,10-phenanthroline-5,6-dione on cellular and virulence aspects of *Scedosporium apiospermum*. Curr. Top. Med. Chem..

[27] McCann M., Santos A.L.S., Silva B.A., Romanos M.T.V., Pyrrho A.S., Devereux M., Kavanagh K., Fichtner I., Kellett A. (2012). In vitro and in vivo studies into the biological activities of 1,10-phenanthroline, 1,10-phenanthroline-5,6-dione and its copper(II) and silver(I) complexes. Toxicol. Res..

[28] Santos A.L.S., Lima A.K.C., Oliveira S.S.C., dos Santos R.F., Devereux M., McCann M., Branquinha M.H., Dutra P.M.L. (2022). Decoding the anti-*Leishmania braziliensis* activity of 1,10-phenanthroline-5,6-dione and its silver- and copper-based complexes: In vitro and in vivo approaches. Eur. J. Med. Chem. Rep..

[29] Reis F.C.G., Borges B.S., Jozefowicz L.J., Sena B.A.G., Garcia A.W.A., Medeiros L.C., Martins S.T., Honorato L., Schrank A., Vainstein M.H. (2019). A novel protocol for the isolation of fungal extracellular vesicles reveals the participation of a putative scramblase in polysaccharide export and capsule construction in *Cryptococcus gattii*. mSphere.

[30] Cirino M.E., Teixeira T.R., da Silva A.M.H., Borges A.C.C., Fukui-Silva L., Wagner L.G., Fernandes C., McCann M., Santos A.L.S., de Moraes J. (2025). Anthelmintic activity of 1,10-phenanthroline-5,6-dione-based metallodrugs. Sci. Rep..

[31] (2017). Reference Method for Broth Dilution Antifungal Susceptibility Testing of Yeasts, 4th ed.

[32] (2020). Epidemiological Cutoff Values for Antifungal Susceptibility Testing, 3rd ed.

[33] Frota H.F., Barbosa P.F., Lorentino C.M.A., Affonso L.R.F., Ramos L.S., Oliveira S.S.C., Souza L.O.P., Abosede O.O., Ogunlaja A.S., Branquinha M.H. (2024). Unveiling the antifungal mechanisms of CTP, a new copper(II)-theophylline/1,10-phenanthroline complex, on drug-resistant non-albicans *Candida* species. Biometals.

[34] Daina A., Michielin O., Zoete V. (2017). SwissADME: A free web tool to evaluate pharmacokinetics, drug-likeness and medicinal chemistry friendliness of small molecules. Sci. Rep..

[35] Lipinski C.A., Lombardo F., Dominy B.W., Feeney P.J. (2001). Experimental and computational approaches to estimate solubility and permeability in drug discovery and development settings. Adv. Drug Deliv. Rev..

[36] Ghose A.K., Viswanadhan V.N., Wendoloski J.J. (1999). A knowledge-based approach in designing combinatorial or medicinal chemistry libraries for drug discovery. 1. A qualitative and quantitative characterization of known drug databases. J. Comb. Chem..

[37] Veber D.F., Johnson S.R., Cheng H.Y., Smith B.R., Ward K.W., Kopple K.D. (2002). Molecular properties that influence the oral bioavailability of drug candidates. J. Med. Chem..

[38] Egan W.J., Merz K.M., Baldwin J.J. (2000). Prediction of drug absorption using multivariate statistics. J. Med. Chem..

[39] Muegge I., Heald S.L., Brittelli D. (2001). Simple selection criteria for drug-like chemical matter. J. Med. Chem..

[40] Daina A., Zoete V. (2016). A BOILED-Egg to predict gastrointestinal absorption and brain penetration of small molecules. ChemMedChem.

[41] Evans B.C., Nelson C.E., Yu S.S., Beavers K.R., Kim A.J., Li H., Nelson H.M., Giorgio T.D., Duvall C.L. (2013). Ex vivo red blood cell hemolysis assay for the evaluation of pH-responsive endosomolytic agents for cytosolic delivery of biomacromolecular drugs. J. Vis. Exp..

[42] Silva L.N., Campos-Silva R., Ramos L.S., Trentin D.S., Macedo A.J., Branquinha M.H., Santos A.L.S. (2018). Virulence of *Candida haemulonii* complex in *Galleria mellonella* and efficacy of classical antifungal drugs: A comparative study with other clinically relevant non-albicans *Candida* species. FEMS Yeast Res..

[43] Guengerich F.P. (2006). Cytochrome P450s and other enzymes in drug metabolism and toxicity. AAPS J..

[44] Serrano I., Verdial C., Tavares L., Oliveira M. (2023). The virtuous *Galleria mellonella* model for scientific experimentation. Antibiotics.

[45] Ikuta K.S., Meštrović T., Naghavi M. (2024). Global incidence and mortality of severe fungal disease. Lancet Infect Dis..

[46] Alexander J.W. (2009). History of the medical use of silver. Surg. Infect.

[47] Barbasz A., Oćwieja M., Walas S. (2017). Toxicological effects of three types of silver nanoparticles and their salt precursors acting on human U-937 and HL-60 cells. Toxicol. Mech. Methods.

[48] Żyro D., Sikora J., Szynkowska-Jóźwik M.I., Ochocki J. (2023). Silver, its salts and application in medicine and pharmacy. Int. J. Mol. Sci..

[49] Padhye L.P., Jasemizad T., Bolan S., Tsyusko O.V., Unrine J.M., Biswal B.K., Balasubramanian R., Zhang Y., Zhang T., Zhao J. (2023). Silver contamination and its toxicity and risk management in terrestrial and aquatic ecosystems. Sci. Total Environ..

[50] Ciardulli M.C., Mariconda A., Sirignano M., Lamparelli E.P., Longo R., Scala P., D’Auria R., Santoro A., Guadagno L., Della Porta G. (2023). Activity and selectivity of novel chemical metallic complexes with potential anticancer effects on melanoma cells. Molecules.

[51] Fabijańska M., Rybarczyk-Pirek A.J., Dominikowska J., Stryjska K., Żyro D., Markowicz-Piasecka M., Szynkowska-Jóźwik M.I., Ochocki J., Sikora J. (2024). Silver complexes of miconazole and metronidazole: Potential candidates for melanoma treatment. Int. J. Mol. Sci..

[52] Mahmoud A.G., Sousa S.A., Guedes da Silva M.F.C., Martins L.M.D.R.S., Leitão J.H. (2024). Antimicrobial activity of water-soluble silver complexes bearing c-scorpionate ligands. Antibiotics.

[53] Kongot M., Reddy D.S., Singh V., Patel R., Singhal N.K., Kumar A. (2020). Physicochemical, in vitro therapeutic activity and biomolecular interaction studies of Mn(II), Ni(II) and Cu(II) complexes tethered with O_2_N_2_ ligand backbone. Spectrochim. Acta A Mol. Biomol. Spectrosc..

[54] Sinha A., Chaudhary R., Reddy D.S., Kongot M., Kurjogi M.M., Kumar A. (2022). ON donor tethered copper (II) and vanadium (V) complexes as efficacious anti-TB and antifungal agents with spectroscopic approached HSA interactions. Heliyon.

[55] Frei A., King A.P., Lowe G.J., Cain A.K., Short F.L., Dinh H., Elliott A.G., Zuegg J., Wilson J.J., Blaskovich M.A.T. (2021). Nontoxic cobalt(III) schiff base complexes with broad-spectrum antifungal activity. Chem. Eur. J..

[56] Mansour A.M., Radacki K. (2020). Antimicrobial properties of half-sandwich Ir(III) cyclopentadienyl complexes with pyridylbenzimidazole ligands. Dalton. Trans..

[57] Mansour A.M., Radacki K. (2020). Experimental and DFT studies of sulfadiazine ‘piano-stool’ Ru(II) and Rh(III) complexes. RSC Adv..

[58] Donnici C.L., Nogueira L.J., Araujo M.H., Oliveira S.R., Magalhães T.F., Lopes M.T., Araújo e Silva A.C., Ferreira A.M., Martins C.V., de Resende Stoianoff M.A. (2014). In vitro studies of the activity of *Dithiocarbamate organoruthenium* complexes against clinically relevant fungal pathogens. Molecules.

[59] Agh-Atabay N.M., Dulger B., Gucin F. (2005). Structural characterization and antimicrobial activity of 1,3-bis(2-benzimidazyl)-2-thiapropane ligand and its Pd(II) and Zn(II) halide complexes. Eur. J. Med. Chem..

[60] Fondjo E.S., Siéwé D.A., Tamokou J.D., Ekom S.E., Djeukoua S.K.D., Doungmo G., Walters M.E., Tsopmo A., Simon P.F.W., Kuiate J.R. (2020). Room temperature synthesis and characterization of novel Bi(III) complex with 2-amino-3-carbomethoxy-4,5,6,7-tetrahydrobenzo[B]thiophene as potential antimicrobial agent. Acta Chim. Slov..

[61] Kuchar J., Rust J., Lehmann C.W., Mohr F. (2020). Silver(I) complexes with camphorsulfonato and phosphine ligands: Structural diversity and antibacterial activity. Inorg. Chem..

[62] Quiles-Melero I., García-Rodríguez J. (2021). Antifúngicos de uso sistémico [Systemic antifungal drugs]. Rev. Iberoam. Micol..

[63] Di L., Kerns E.H., Carter G.T. (2009). Drug-like property concepts in pharmaceutical design. Curr. Pharm. Des..

[64] Silva V., Gil-Martins E., Silva B., Rocha-Pereira C., Sousa M.E., Remião F., Silva R. (2021). Xanthones as P-glycoprotein modulators and their impact on drug bioavailability. Expert Opin. Drug Metab. Toxicol..

[65] Rossato L., Loreto É.S., Zanette R.A., Chassot F., Santurio J.M., Alves S.H. (2016). In vitro synergistic effects of chlorpromazine and sertraline in combination with amphotericin B against *Cryptococcus neoformans* var. grubii. Folia Microbiol..

[66] Iyer K.R., Revie N.M., Fu C., Robbins N., Cowen L.E. (2021). Treatment strategies for cryptococcal infection: Challenges, advances and future outlook. Nat. Rev. Microbiol.

[67] Xu C.Y., Zhu H.M., Wu J.H., Wen H., Liu C.J. (2014). Increased permeability of blood-brain barrier is mediated by serine protease during *Cryptococcus* meningitis. J. Int. Med. Res..

[68] Woo Y.H., Martinez L.R. (2021). *Cryptococcus neoformans*-astrocyte interactions: Effect on fungal blood brain barrier disruption, brain invasion, and meningitis progression. Crit. Rev. Microbiol..

[69] Martignoni M., Groothuis G.M., de Kanter R. (2006). Species differences between mouse, rat, dog, monkey and human CYP-mediated drug metabolism, inhibition and induction. Expert Opin. Drug Metab. Toxicol..

[70] Albadry M., Küttner J., Grzegorzewski J., Dirsch O., Kindler E., Klopfleisch R., Liska V., Moulisova V., Nickel S., Palek R. (2024). Cross-species variability in lobular geometry and cytochrome P450 hepatic zonation: Insights into CYP1A2, CYP2D6, CYP2E1 and CYP3A4. Front. Pharmacol..

[71] Qian J., Guo Y., Xu Y., Wang X., Chen J., Wu X. (2023). Combination of micelles and liposomes as a promising drug delivery system: A review. Drug Deliv. Transl. Res..

[72] Lubran M.M. (1989). Hematologic side effects of drugs. Ann. Clin. Lab. Sci..

[73] Singh U., Dar M.M., Anayutullah S., Alam H., Manzoor N., Al-Thabaiti S.A., Hashmi A.A. (2015). Design and synthesis of Co(II) and Cu(II) complexes of a dendrimeric chelate: Promising anticandidal potential of chelotherapeutic agents. J. Coord. Chem..

[74] Farag M.R., Alagawany M. (2018). Erythrocytes as a biological model for screening of xenobiotics toxicity. Chem. Biol. Interact..

[75] Marin M., Fernández A., Sánchez-Yagüe J., Cabezas J., Llanillo M. (1990). Changes in the phospholipid and fatty acid composition in normal erythrocytes from sheep of different ages. Aminophospholipid organization in the membrane bilayer. Biochimie.

[76] Calabrese E., Williams P., Moore G. (1983). An evaluation of the dorset sheep as a predictive animal model for the response of glucose-6-phosphate dehydrogenase-deficient human erythrocytes to a proposed systemic toxic ozone intermediate, methyl oleate ozonide. Ecotoxicol. Environ. Saf..

[77] Mikulak E., Gliniewicz A., Przygodzka M., Solecka J. (2018). *Galleria mellonella* L. as model organism used in biomedical and other studies. Przegl. Epidemiol..

[78] Giammarino A., Bellucci N., Angiolella L. (2024). *Galleria mellonella* as a model for the study of fungal pathogens: Advantages and disadvantages. Pathogens.

[79] Gallorini M., Marinacci B., Pellegrini B., Cataldi A., Dindo M.L., Carradori S., Grande R. (2024). Immunophenotyping of hemocytes from infected *Galleria mellonella* larvae as an innovative tool for immune profiling, infection studies and drug screening. Sci. Rep..

[80] Mesa-Arango A.C., Scorzoni L., Zaragoza O. (2012). It only takes one to do many jobs: Amphotericin B as antifungal and immunomodulatory drug. Front. Microbiol..

[81] Kelly J., Kavanagh K. (2011). Caspofungin primes the immune response of the larvae of *Galleria mellonella* and induces a non-specific antimicrobial response. J. Med. Microbiol..

[82] Smith D.F.Q., Casadevall A. (2021). Fungal immunity and pathogenesis in mammals versus the invertebrate model organism *Galleria mellonella*. Pathog. Dis..

[83] Hurlow J., Bowler P.G. (2022). Acute and chronic wound infections: Microbiological, immunological, clinical and therapeutic distinctions. J. Wound Care.

[84] Stielow M., Witczyńska A., Kubryń N., Fijałkowski Ł., Nowaczyk J., Nowaczyk A. (2023). The bioavailability of drugs—The current state of knowledge. Molecules.

[85] Louis-Maerten E., Rodriguez Perez C., Cajiga R.M., Persson K., Elger B.S. (2024). Conceptual foundations for a clarified meaning of the 3Rs principles in animal experimentation. Anim. Welf..

